# Sympathetic Overactivity in Chronic Kidney Disease: Consequences and Mechanisms

**DOI:** 10.3390/ijms18081682

**Published:** 2017-08-02

**Authors:** Jasdeep Kaur, Benjamin E. Young, Paul J. Fadel

**Affiliations:** Department of Kinesiology, University of Texas at Arlington, Arlington, TX 76019, USA; jasdeep.kaur@uta.edu (J.K.); Ben.young@mavs.uta.edu (B.E.Y.)

**Keywords:** nitric oxide, asymmetric dimethylarginine, blood pressure, oxidative stress, sympathetic outflow, hypertension, muscle sympathetic nerve activity, cardiovascular disease, angiotensin II

## Abstract

The incidence of chronic kidney disease (CKD) is increasing worldwide, with more than 26 million people suffering from CKD in the United States alone. More patients with CKD die of cardiovascular complications than progress to dialysis. Over 80% of CKD patients have hypertension, which is associated with increased risk of cardiovascular morbidity and mortality. Another common, perhaps underappreciated, feature of CKD is an overactive sympathetic nervous system. This elevation in sympathetic nerve activity (SNA) not only contributes to hypertension but also plays a detrimental role in the progression of CKD independent of any increase in blood pressure. Indeed, high SNA is associated with poor prognosis and increased cardiovascular morbidity and mortality independent of its effect on blood pressure. This brief review will discuss some of the consequences of sympathetic overactivity and highlight some of the potential pathways contributing to chronically elevated SNA in CKD. Mechanisms leading to chronic sympathoexcitation in CKD are complex, multifactorial and to date, not completely understood. Identification of the mechanisms and/or signals leading to sympathetic overactivity in CKD are crucial for development of effective therapeutic targets to reduce the increased cardiovascular risk in this patient group.

## 1. Introduction

More than 26 million people in the United States suffer from chronic kidney disease (CKD) with thousands of new cases diagnosed each year [1]. CKD has poor prognosis and health outcomes with very high health care costs. In the US alone, the cost of treatment for CKD has exceeded 49 billion dollars per year [2,3]. There are five stages of CKD defined by progressive decreases in renal function as quantified by estimated glomerular filtration rate (eGFR) and/or kidney damage (proteinuria, albuminuria). Stage 1 CKD constitutes kidney damage with normal or reduced eGFR (≥90 mL/min/1.73 m^2^). Stages 2, 3 and 4 show progressive renal dysfunction with an eGFR of 60–89, 30–59 and 15–29 mL/min/1.73 m^2^, respectively. Stage 5 CKD is renal failure or end-stage renal disease which is characterized by eGFR of <15 mL/min/1.73 m^2^ or dialysis. Moderate to severe CKD (stage 3–5) is associated with a significantly higher risk of cardiovascular (CV) morbidity and mortality [4,5]. In fact, over 50% of deaths in renal failure patients occur due to myocardial infarction, stroke, heart failure, and sudden cardiac death, the latter accounting for 25% of deaths in these patients alone [6,7,8]. There is growing evidence that sympathetic overactivity, a characteristic feature of CKD, is one of the major mechanisms leading to higher CV risk in this patient population [9,10]. This brief review will discuss some of the consequences of chronically elevated sympathetic nerve activity (SNA) and highlight some of the potential pathways contributing to elevated SNA in CKD.

**Figure 1:**
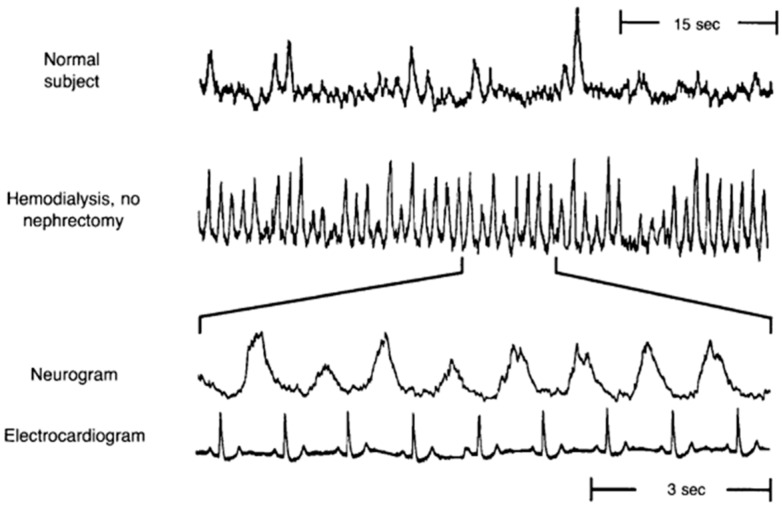
Original record of muscle sympathetic nerve activity (MSNA) in a normal subject and a renal failure patient on hemodialysis, demonstrating significantly greater resting MSNA in the dialysis patient. The bottom two panels display a portion of the neurogram and simultaneous electrocardiogram from the patient on hemodialysis showing that the renal failure patient has a burst of MSNA with every cardiac cycle. (Modified from Converse et al. [11] with permission).

**Figure 2:**
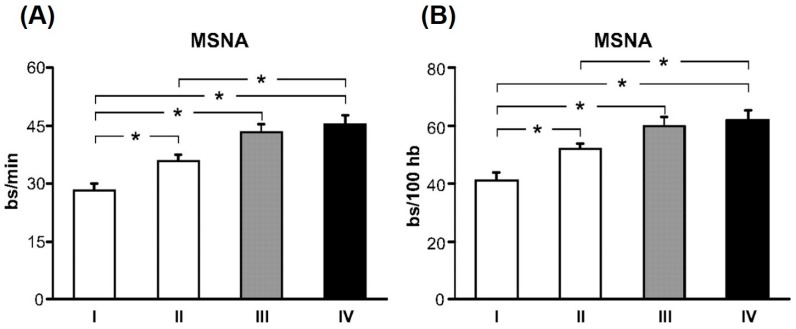
Muscle sympathetic nerve activity (MSNA) expressed as burst frequency (bursts (bs)/min, **A**) and burst incidence (bs/100 heartbeats (hb), **B**) in subjects grouped according to their estimated glomerular filtration rate (eGFR), with the highest quartile of eGFR in group I and lowest quartile of eGFR in group IV. With decreasing eGFR, there is a significant and progressive increase in resting MSNA in chronic kidney disease (CKD) patients. * *p* < 0.05 significant difference between quartiles. (From Grassi et al. [13] with permission).

A multitude of studies have shown elevated SNA in CKD [11,12,13,14,15]. Early observations of exaggerated SNA in renal failure patients came from studies showing significantly elevated plasma norepinephrine, the primary neurotransmitter released by sympathetic nerves [16,17]. Several studies have also used the technique of microneurography to directly record from post-ganglionic sympathetic nerve fibers innervating blood vessels that supply skeletal muscle (i.e., muscle sympathetic nerve activity (MSNA)). Converse et al. [11] was the first to report elevated resting MSNA in renal failure patients on hemodialysis (Figure 1). However, of note, sympathoexcitation is not confined to renal failure, but is also detectable in earlier stages of CKD [13,15,18,19]. Indeed, Grassi et al. [13] studied mild-to-moderate CKD patients who were divided into four groups based on their eGFR, with highest quartile of eGFR (95.4 ± 1.6 mL/min/1.73 m^2^) in group I and lowest quartile of eGFR (31.4 ± 1.8 mL/min/1.73 m^2^) in group IV. There was a significant and progressive increase in resting MSNA from the highest quartile to the lowest quartile of eGFR (Figure 2) [13]. Other studies have reported elevated MSNA even in patients with polycystic kidney disease and no impairment in renal function [18,19]. Collectively, these findings demonstrate two important points: (1) SNA is augmented early in CKD; and (2) SNA increases progressively with declining renal function.

## 2. Consequences of Sympathetic Overactivity

Sympathetic overactivity is associated with various diseases and accelerates the progression of cardiovascular, metabolic and renal pathology [20,21,22]. The most common detrimental consequence of elevated SNA is hypertension [23,24,25], which is observed in 80% of CKD patients [26,27]. Hypertension, in turn, leads to structural and functional abnormalities such as left ventricular hypertrophy, increased vascular smooth muscle cell hyperplasia and hypertrophy, and endothelial dysfunction [28,29,30]. More importantly, chronic sympathoexcitation independent of hypertension has detrimental effects. For example, administration of sub-hypertensive doses of norepinephrine has been shown to cause myocardial cell hypertrophy and left ventricular hypertrophy [31,32,33]. Moreover, elevated SNA without increases in blood pressure has been shown to cause vascular smooth muscle cell hypertrophy and proliferation [34,35]. In addition, other deleterious effects of elevated SNA, independent of increased blood pressure, include, but are not limited to, exaggerated coronary vasoconstriction [36], arrythmogenicity [37], impaired renal function [38], glomerular podocyte injury [39], metabolic impairment [40], increased arterial stiffness [41,42,43], endothelial dysfunction [44,45,46] and subsequent development of atherosclerosis [47,48], all of which lead to increased risk of CV events.

On the other hand, reducing SNA without any lowering of blood pressure has been shown to be protective, in particular to the kidney [20]. In this regard, treatment of partially nephrectomized rats (an animal model of CKD) with non-hypotensive doses of the sympatholytic agent moxonidine, resulted in reduced glomerulosclerosis and urinary albumin excretion compared to untreated nephrectomized animals [20]. Furthermore, low doses of moxonidine that did not change blood pressure were shown to elicit an antialbuminuric effect in diabetic patients [49]. Collectively, these data provide strong evidence that sympathetic overactivity, independent of increases in blood pressure, can cause detrimental cardiovascular, metabolic, and renal effects. Although there is clear evidence of sympathetic overactivity in CKD, the mechanisms leading to chronic sympathoexcitation in CKD are complex, multifactorial and not completely understood. The remainder of this review will focus on some of the potential mechanisms contributing to elevated SNA in CKD.

## 3. Mechanisms of Sympathetic Overactivity in Chronic Kidney Disease (CKD)

### 3.1. Renin–Angiotensin System

It is well known that the renin–angiotensin system is activated in CKD. Renin is secreted from the kidney and converts angiotensinogen to angiotensin I, which in turn is converted to angiotensin II (Ang II) by angiotensin-converting enzyme (ACE). Activation of renin–angiotensin system increases renin secretion ultimately leading to high circulating plasma concentrations of Ang II, a common feature of CKD [50,51,52]. Ang II is a potent vasoconstrictor with a multitude of peripheral and central actions as previously described in detail (for review see [38,53,54]). Briefly, in addition to causing direct peripheral vasoconstriction, Ang II also modulates peripheral SNA by potentiating norepinephrine release from sympathetic nerve terminals. Ang II also plays an important role in regulating sympathetic outflow from the brainstem. For example, microinjection of Ang II into the rostral ventrolateral medulla (RVLM) activates vasomotor sympathetic neurons resulting in elevated SNA [55,56,57] while microinjection of an Ang II receptor blocker, losartan, into the RVLM causes sympathoinhibition [57,58]. These findings and others [53,59,60] provide clear evidence for a direct role of Ang II in regulating central sympathetic outflow. Although Ang II receptor blockers and ACE inhibitors are a first-line choice of treatment in CKD patients, chronic treatment with these drugs only reduces MSNA but does not normalize it [15,16]. In other words, MSNA in CKD patients following chronic treatment with Ang II receptor blockers and ACE inhibitors is still higher than in healthy individuals. Therefore, mechanisms other than the renin–angiotensin system are also involved in causing sympathetic overactivity in CKD.

### 3.2. Renal Afferents

The kidneys are richly innervated by chemoreceptors and baroreceptors which send feedback to the brain to regulate sympathetic outflow and systemic blood pressure. Evidence from both animal and human studies indicates that neural signals originating from the kidney play a role in increasing sympathetic outflow in CKD [61,62]. Rats subjected to 5/6th nephrectomy, an animal model of CKD, develop hypertension with elevated norepinephrine turnover in various hypothalamic nuclei involved in the regulation of sympathetic outflow. Selective removal of afferent nerves (dorsal rhizotomy) in these animals prevents both the development of hypertension and increase in norepinephrine turnover in hypothalamic nuclei in the brain [61]. Furthermore, renal injury without a reduction in renal function also leads to augmented sympathetic outflow. Animal studies have shown that renal injury via intrarenal phenol injection, which does not decrease renal function, causes increases in renal SNA, plasma norepinephrine concentrations, blood pressure, and norepinephrine secretion in the posterior hypothalamus [62,63,64]. In addition, ligands such as urea and adenosine, which are elevated in CKD, can stimulate renal nerves, also contributing to increases in SNA [65,66]. Renal ischemia enhances adenosine production, which not only stimulates renal afferent nerves but also causes vasoconstriction of afferent arterioles leading to reduced GFR [67]. Interestingly, Hausberg et al. [68] observed that MSNA in renal transplant patients with intact native kidneys was similar to that in dialysis patients (stage 5 CKD). However, transplant patients who underwent bilateral native kidney nephrectomy exhibited a significant reduction in MSNA, to values not that different from control subjects. Thus, signals arising from the native kidney can contribute to heightened MSNA in renal disease. Moreover, recent studies have suggested reduced renalase levels as a potential contributing factor in elevated SNA in CKD [69,70,71]. Renalase is a monoamine oxidase produced by the kidneys that circulates in the blood in its inactive form prorenalase [70]. Once activated, renalase degrades catecholamines and can decrease blood pressure [70,72]. CKD and renal failure patients have significantly reduced levels of renalase, which would lead to less breakdown of catecholamines and contribute to the higher SNA in these patients [70].

### 3.3. Nitric Oxide Pathway

Another potential mechanism for chronic sympathoexcitation in CKD is reduced nitric oxide (NO) bioavailability. NO is produced by nitric oxide synthase (NOS) during oxidation of l-arginine to l-citrulline [73]. There are three isoforms of NOS: endothelial NOS (eNOS), neuronal NOS (nNOS) and inducible NOS (iNOS). While eNOS and nNOS are constitutively expressed in all cells and contribute to the regulation of vascular tone and blood pressure, iNOS is activated by macrophages and cytokines during inflammation. NO produced by eNOS diffuses into smooth muscle cells and causes vasodilation via stimulation of guanylate cyclase. Indeed, systemic NOS inhibition in healthy individuals causes an immediate increase in arterial blood pressure by reducing NO and endothelium-mediated vasodilation [74,75,76]. In addition to its action as a vasodilator, NO also plays a key role in maintaining vascular homeostasis by inhibiting platelet aggregation, atherogenesis, smooth muscle cell proliferation and leukocyte adhesion to the endothelium [77,78]. Thus, endothelium-derived NO not only regulates vascular tone and thereby, arterial blood pressure but it also plays an essential role in maintaining a healthy vasculature. While the role of peripheral NO in endothelium-mediated vasodilation and blood pressure regulation is well known, much less appreciated is the potential central effect of NO.

There is increasing evidence that suggests NO is a key signaling molecule involved in regulation of sympathetic outflow from the brainstem. Shapoval et al. [79] performed the first in vivo study to demonstrate a direct effect of central NO on sympathetic outflow. They showed that microinjection of the NOS inhibitor N^G^-monomethyl l-arginine (l-NMMA) directly into the RVLM of anesthetized animals increased renal SNA and consequently, blood pressure, while microinjections of l-arginine and sodium nitroprusside (NO donor) reduced renal SNA and blood pressure [79]. Microinjection of l-NMMA into the nucleus tractus solitarius (NTS) showed similar changes in renal SNA and blood pressure [80]. Tagawa and colleagues [81] used rat brainstem slices to show that l-arginine causes a dose-dependent increase in neuronal activity of ~40% of NTS neurons, and this response is attenuated by l-NMMA. Hemoglobin, which endogenously binds to NO, blocks the increase in neuronal activity evoked by l-arginine, suggesting that NO diffuses out into the extracellular space to excite the adjacent neurons from which the neural activity was measured. They further extended their findings by using methylene blue, a guanylate cyclase blocker, to investigate whether these effects of l-arginine were mediated by cyclic guanosine monophosphate (cGMP). Methylene blue inhibits the increase in neuronal activity in the NTS elicited by both l-arginine and sodium nitroprusside. These data indicate that NO produced from l-arginine in the NTS neurons diffuses out to nearby target neurons where it increases neuronal activity through cGMP. Other animal studies involved intravenous infusion, interacisternal injection or microinjection of NOS inhibitors into the paraventricular nucleus (PVN) and the NTS, demonstrating acute increases in renal SNA and blood pressure [74,80,82]. Furthermore, overexpression of NOS in the RVLM via adenovirus transfection results in elevated NO levels in the RVLM, which reduced urinary norepinephrine excretion along with a lowering of blood pressure [83,84]. Together, these studies indicate that centrally-derived NO is a key signaling molecule involved in the tonic restraint of sympathetic outflow from the brainstem.

Initial studies performed to extrapolate these findings from direct central injections to systemic infusions of NOS inhibitors observed a reduction in SNA. Indeed, when NOS inhibitors were infused systemically in animals and humans, there was a rapid and large increase in blood pressure along with a decrease in SNA [85,86]. The rapid increase in blood pressure occurred due to inhibition of NO-mediated, endothelium-dependent vasodilation in the peripheral vasculature. This increase in blood pressure activated the arterial baroreflex resulting in a reflex-mediated decrease in SNA. Thus, baroreflex-mediated reductions in SNA masked any increases in sympathetic outflow that might have occurred due to reduced central NO. To eliminate the influence of the arterial baroreflex, the effect of systemic NOS inhibition was compared between barointact and barodenervated animals [74,87]. Administration of the NOS inhibitor l-nitroarginine methyl ester (l-NAME) in barointact animals caused a biphasic response in renal SNA, an initial decrease followed by an increase in renal SNA. In barodenervated animals, NOS inhibition caused a progressive and significant increase in renal SNA uncovering the role of central NO in restraining sympathetic outflow. Our laboratory has also provided evidence for NO in central sympathetic control in humans. To overcome the confounding inhibitory influence of the arterial baroreflex on SNA, we directly measured skin SNA, which is not under baroreceptor control. Systemic l-NAME infusion in healthy adults caused progressive and sustained increases in skin SNA [88].

**Figure 3:**
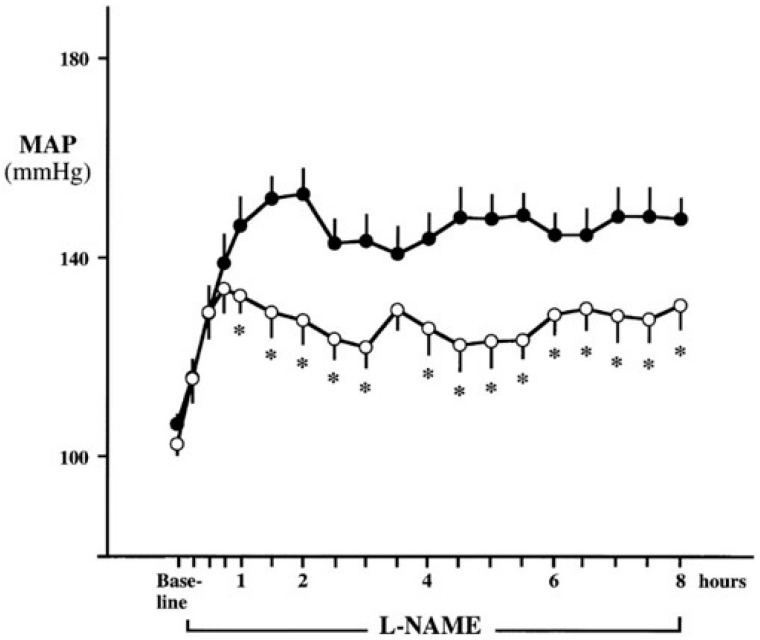
Mean arterial pressure (MAP) responses during 8-h continuous systemic infusion of the nitric oxide synthase (NOS) inhibitor l-nitroarginine methyl ester (l-NAME) in rats with sympathectomy (open circles) and without sympathectomy (closed circles). Although no difference was observed in the initial increase in MAP (removal of endothelium-mediated dilation), sympathectomy attenuated the sustained hypertensive response to l-NAME, demonstrating a sympathetic contribution to the blood pressure raising effects of systemic NOS inhibition. * *p* < 0.05 vs. control. (From Sander et al. [75] with permission).

Another consideration with systemic infusion of NOS inhibitors to examine effects of central NO on SNA is the time needed for the inhibitors to cross the blood brain barrier. In this regard, systemic infusion of l-NAME in animals with and without sympathectomy showed that both groups had a similar increase in blood pressure during the first hour of infusion primarily due to inhibition of peripheral endothelial NO production. Importantly, blood pressure in the sympathectomized animals after 8 h and after 6 days of l-NAME infusion was significantly lower than the control group, indicating a delayed but significant role of the sympathetic nervous system in the blood pressure-raising effect of NOS inhibition (Figure 3) [75]. In agreement with a time delay in sympathetic activation via systemic NOS inhibition, when phentolamine (α-adrenergic blocker) was infused immediately and 90 min after l-NAME infusion in humans, there was little effect on the initial hypertensive response but phentolamine significantly attenuated the subsequent further increase in blood pressure [76]. These results indicate that the initial increase in blood pressure occurred due to diminished peripheral NO causing inhibition of endothelium-mediated vasodilation while the later increase in blood pressure was caused by sympathetic adrenergic vasoconstriction. This would also explain the results observed in initial studies where NOS inhibition caused a reduction in SNA, as SNA was only measured for 40 min after l-NAME infusion [86], which may not have been long enough to observe central effects of systemic NOS inhibition. Taken together, these data indicate the role of central NO in tonically restraining sympathetic outflow from the brainstem in healthy rodents and humans. Thus, it is plausible that in CKD, reduced central NO concentrations potentially contribute to chronically elevated SNA in these patients.

One of the major mechanisms causing reduced NO concentrations in CKD is elevated levels of asymmetric dimethylarginine (ADMA), the primary endogenous NOS inhibitor [89]. Plasma concentrations of ADMA are significantly elevated in mild CKD and increase progressively as renal function declines [14,90]. Importantly, numerous studies have shown ADMA to be a strong, independent predictor of future CV risk in CKD patients [10,91,92,93,94,95,96]. In fact, Zoccali et al. [94] reported that in renal failure patients, plasma ADMA concentrations are the second strongest predictor of all-cause and CV mortality (after age). ADMA concentrations also predict CV risk and mortality in earlier stages of CKD. For example, in a large cohort of stage 3 to 4 CKD patients, elevated ADMA was shown to have a strong association with CV disease and a modest association with all-cause and CV mortality [97]. While it is clear that high ADMA levels are associated with CV risk, to date the majority of work with ADMA has been correlational in nature with a focus on the well-known vascular endothelial properties of NO. Given the increasing functional evidence described above that indicates NO is also a key signaling molecule involved in the tonic restraint of central sympathetic outflow, a role for ADMA in contributing to sympathetic overactivity in CKD warrants consideration.

**Figure 4:**
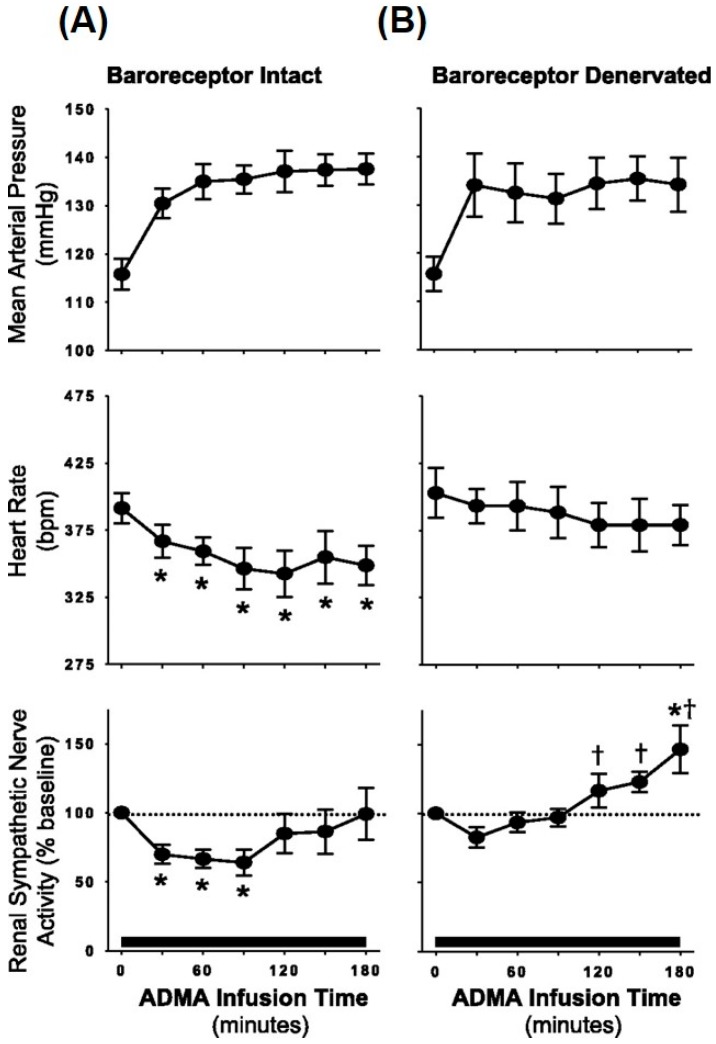
Average mean arterial pressure, heart rate, and renal sympathetic nerve activity during continuous systemic infusion of asymmetric dimethylarginine (ADMA; an endogenous NOS inhibitor) in baroreceptor-intact (**A**) and baroreceptor-denervated animals (**B**). In comparison with barointact animals, barodenervated animals show a significant increase in renal SNA indicating frank sympathoexcitation in response to systemic ADMA infusion. * *p* < 0.05 vs. baseline; ^†^ *p* < 0.05 vs. baroreceptor intact. (From Augutyniak et al. [87] with permission.)

**Figure 5:**
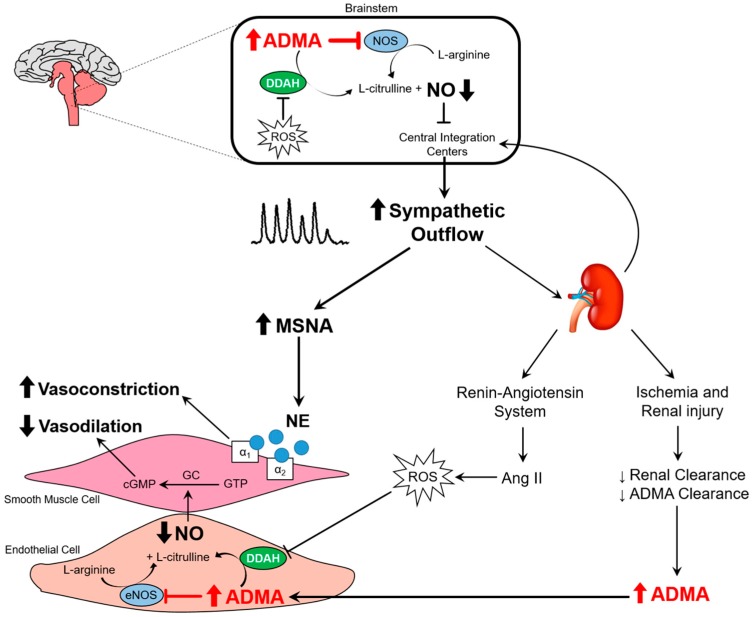
Schematic illustration depicting the effects of elevated ADMA in the brainstem and the peripheral circulation. Reduced dimethylarginine dimethylaminohydrolase (DDAH) activity and renal clearance of ADMA leads to elevated plasma ADMA concentrations in chronic kidney disesase (CKD). Elevated ADMA in the brainstem inhibits NOS and reduces central nitric oxide (NO) production, contributing to higher central sympathetic outflow. This greater SNA results in peripheral vasoconstriction. When prolonged, the sympathetic overactivity leads to a host of other deleterious consequences as outlined in the text of the review. In the periphery, elevated ADMA inhibits NOS and decreases NO, thereby reducing endothelium-mediated vasodilation. Greater sympathetically-mediated vasoconstriction and lower endothelium-mediated vasodilation contributes to increased vascular tone and higher blood pressure. ROS, reactive oxygen species; eNOS, endothelial nitric oxide synthase; NE, norepinephrine. ⱶ- denotes inhibition. (See text for further details).

ADMA is produced in all cell types and can pass the blood–brain barrier [98,99]. It is produced by post-translational methylation of proteins via the enzyme arginine methyltransferase type I [95,99] and it is either metabolized by the dimethylarginine dimethylaminohydrolase (DDAH) enzyme or excreted by the kidneys [99,100]. Although reduced renal clearance of ADMA in CKD contributes to elevated plasma levels, the major pathway for elimination of ADMA is its metabolism by DDAH. It is estimated that the human body produces about 300 µmol of ADMA every day, of which 250 µmol is metabolized by DDAH and only a small amount is excreted by the kidneys [101]. In terms of sympathetic activation and ADMA, Augustyniak et al. [87] investigated the potential effects of ADMA on sympathetic outflow by systemic ADMA infusions in barointact and barodenervated animals. In barointact animals, ADMA infusion caused an initial decrease in renal SNA followed by an increase, causing renal SNA to go back to baseline values. In contrast, in barodenervated animals, systemic ADMA infusion caused a frank sympathoexcitation as indicated by ~50% increase in renal SNA at the end of ADMA infusion (Figure 4). Thus, elevated ADMA levels can cause inhibition of central NOS, thereby reducing NO bioavailability and increasing SNA. These data support the idea that elevated ADMA reduces central NO in CKD patients, potentially contributing to elevated sympathetic outflow from the brainstem (Figure 5). Along these lines, ADMA levels and resting muscle SNA were inversely related to eGFR in stage 2 to 4 CKD patients such that patients with higher ADMA levels had higher resting SNA and lower renal function (i.e., lower eGFR) [14].

### 3.4. Oxidative Stress

CKD patients are reported to have higher oxidative stress [102,103,104,105,106], which could be another potential mechanism that results in chronic sympathoexcitation in CKD. Oxidative stress in the central nervous system has an important role in regulating sympathetic outflow from the brainstem [107]. Reactive oxygen species (ROS) such as superoxide ion, hydroxyl radical and hydrogen peroxide play an important role as intracellular messengers however, overproduction of ROS can be harmful. Various animal studies have indicated that elevated oxidative stress in the brain contributes to enhanced central sympathetic outflow, either directly or by scavenging NO [107,108,109,110,111]. Elevated levels of Ang II also contribute to overproduction of ROS as Ang II is a potent activator of nicotinamide adenine dinucleotide phosphate (NAD(P)H) oxidase, the primary source of superoxide [112]. Reductions in oxidative stress in the brain using the superoxide dismutase (SOD; an enzyme that catalyzes superoxide ion) mimetic tempol or overexpression of SOD reverses the elevated central sympathetic outflow [113,114,115,116]. In addition, tempol administration through drinking water and systemic tempol infusion have both been shown to normalize SNA and reduce neuronal activity of presympathetic neurons in the PVN and the RVLM [117,118]. These studies provide clear evidence that oxidative stress can cause chronic sympathoexcitation. In addition to its direct effects, elevated oxidative stress also inhibits DDAH, the primary mechanism for breakdown of ADMA [96,119]. This may contribute to ADMA-induced increases in central sympathetic activation. Collectively, these studies indicate that oxidative stress is one of the mechanisms that increases sympathetic outflow from the brainstem and may be involved in elevated SNA in CKD.

## 4. Therapeutic Strategies

Sympathetic overactivity is a hallmark of CKD and therapeutic strategies to reduce this heightened sympathetic activation are needed. In this review we have identified some of the potential pathways contributing to elevated SNA in CKD that warrant consideration when discussing therapeutic targets to reduce SNA in this high risk population. Ang II receptor blockers and ACE inhibitors have been shown to lower resting MSNA and blood pressure but neither normalizes MSNA in CKD and thus, other therapeutic strategies are needed [15,16]. Sympatholytic agents such as moxonidine have been shown to be effective in reducing MSNA in patients [49] however, the side-effects limit their clinical application [120,121,122]. Statins are another potential therapeutic strategy that has been shown to reduce SNA and oxidative stress in addition to lowering cholesterol [123,124]. In fact, statins have been shown to downregulate Ang II receptors and upregulate nNOS in the brain [124]. Even short-term statin therapy has been shown to reduce sympathetic overactivity [125,126], making it a potentially beneficial therapy for CKD patients. Indeed, statin therapy in predialysis CKD patients reduced MSNA [127], delayed the start of dialysis [128], and decreased the risk of CV events and all-cause mortality [129,130]. In addition, reduction of oxidative stress is another potential mechanism for decreasing sympathetic overactivity in CKD. Although numerous animal studies have shown significant reductions in SNA after reducing oxidative stress, studies in patients have provided mixed results [131,132,133,134]. This could be, in part, due to the antioxidant used in the study (vitamin C vs. vitamin E), the duration of treatment (acute vs. chronic treatment), the dose of antioxidant and the efficacy of antioxidant in reducing oxidative stress. Furthermore, pioglitazone treatment may also be beneficial in CKD as it has been reported to reduce circulating ADMA levels [135,136]. Reductions in plasma ADMA concentrations can increase central NO and inhibit sympathetic outflow from the brainstem. Thus, pioglitazone treatment could be a promising therapy for reducing ADMA and sympathetic overactivity in CKD. In general, further research is warranted to identify viable pharmacological therapies to reduce sympathetic overactivity in CKD along with its deleterious consequences.

Aside from the aforementioned pharmacological strategies, there are therapeutic interventions such as renal denervation and carotid baroreflex stimulation that may also be used to reduce SNA in CKD. Renal denervation has been primarily utilized to treat resistant hypertension. Several studies in hypertensive patients and experimental animal disease models showed a significant reduction in blood pressure following renal denervation [137,138,139,140,141,142,143]. Interestingly, in a large controlled clinical trial (SYMPLICITY HTN-3), although hypertensive patients had a reduction in blood pressure following renal denervation, this decrease was not different from a sham control group [144]. Since bilateral nephrectomy in hemodialysis patients has been shown to reduce resting MSNA [11,68], renal denervation might be beneficial for the CKD population. Indeed, pilot studies have shown promising results: besides reducing blood pressure [145,146], renal denervation has also been shown to lower renin production, enhance GFR and reduce albuminuria [147,148]. Nevertheless, despite the advantages, some factors must be considered before performing renal denervation in CKD patients such as: contrast-induced nephropathy in CKD, non-optimal diameter of renal artery and complications due to low renal blood flow [146,149]. Another therapeutic intervention to potentially reduce sympathetic overactivity in CKD patients is carotid baroreceptor stimulation. With this intervention, a device is surgically implanted to chronically stimulate carotid baroreceptors. This technique has been shown to cause sympatho-inhibition and reduce blood pressure in hypertensive patients [150,151,152]. However, further studies are required to investigate the potential beneficial effects of chronic carotid baroreflex stimulation in CKD patients.

## 5. Conclusions

Chronic sympathoexcitation is a major contributor to increased CV risk and mortality in CKD. Despite recent advances in research, our understanding of the mechanisms causing sympathetic overactivity in CKD is still incomplete. As discussed in this review, various studies have proposed a role for the renin–angiotensin system, renal afferent stimulation, reduced NO concentrations due to elevated ADMA, and increased oxidative stress in contributing to chronic sympathoexcitation in CKD. Further research is needed to better clarify the individual and interactive roles of each of the aforementioned pathways in contributing to sympathetic overactivity in CKD patients.

## Abbreviations

ACE	Angiotensin converting enzyme
ADMA	Asymmetric dimethylarginine
bs	Bursts
cGMP	Cellular guanosine monophosphate
CKD	Chronic kidney disease
CV	Cardiovascular
DDAH	Dimethylarginine dimethylaminohydrolase
eGFR	Estimated glomerular filtration rate
eNOS	Endothelial nitric oxide synthase
hb	Heartbeats
iNOS	Inducible nitric oxide synthase
l-NAME	l-N^G^-nitroarginine methyl ester
l-NMMA	N^G^-monomethyl l-arginine
MSNA	Muscle sympathetic nerve activity
NAD(P)H	Nicotinamide adenine dinucleotide phosphate
NE	Norepinephrine
NO	Nitric oxide
NOS	Nitric oxide synthase
nNOS	Neuronal nitric oxide synthase
NTS	Nucleus tractus solitarius
PVN	Paraventricular nucleus
RVLM	Rostral ventrolateral medulla
SNA	Sympathetic nerve activity
SOD	Superoxide dismutase
